# Lived experience narrative: My journey with schizophrenia

**DOI:** 10.4102/sajpsychiatry.v30i0.2259

**Published:** 2024-04-30

**Authors:** Name withheld by request

In the beginning, it felt very unreal, like I was in a fantasy world. I knew something was wrong when I was first diagnosed because my mother and brother were acting very strangely towards me. But I could not finger point what was wrong with me. It is only when I came to hospital that the doctor explained it to me. People with schizophrenia need to come to an acceptance that we have got what the doctor says we have. The problem is, I think people with schizophrenia are not informed properly on what is happening so that we can identify in our minds that these are the symptoms of the illness. We are ill-informed so we act on an impulse. Like, for example, we get angry or try to hurt ourselves when people are shouting at us.

The main important thing that I wish doctors and others treating schizophrenia were talking about is the feeling of isolation that people with schizophrenia have. Isolation, isolation, isolation. I can not stress that enough. When a person is certified, or is diagnosed, they think that ‘normal’ people do not understand what they are going through. You feel like the whole world is over. Sometimes you think the world is out to get you, you think the people with whom you are living with are negative towards you. For me that causes anxiety, which if left untreated, leads to depression. Depression, if left untreated, causes suicidal attempts or anger outbursts, and then anger, which lasts for a long time, worsens schizophrenia. That is based on what I am going through, not necessarily what other people are going through. I do think a lot of other patients feel the same thing because people with schizophrenia tend to bottle up everything inside. The main thing that I think will help people with schizophrenia and similar illnesses is socialising.

When I am with professionals, at first, I tend to think, well, I must listen to them. But they do not know what I am going through. Because you do not live with an illness like me. Like, if I see my friend, I shake his hand, I give him a hug, I ask him, how are you? If I say, you know what, I was feeling depressed and I was in anxiety, then we talk. Whereas in a professional environment, I might think, why am I talking to these doctors? They do not know what the illness feels like. So how would they understand? They just see me based on what they learned at university. I am not saying this to stigmatise the doctors. As time goes by during treatment, we tend to grow an understanding and acceptance that the professionals are there to help us and we develop trust but it is hard if the doctor keeps changing.

Another example, you might get people who are using drugs and who are not totally open with the doctor because they think the doctor is going to judge them. The doctor is going to say, you are on drugs, I am going to give you a higher dose of injection. Not understanding that they are actually trying to help them, instead they think that the doctor is just there to make their life miserable. There is a way that they could work with people who have schizophrenia. Nobody can force a person to do something that they do not want to do. So, the doctors need to help the person with schizophrenia find a vision for their life. We need to feel like we belong somewhere, where we can talk about our problems or laugh or cry or just express emotions, especially by talking with other people who have the same illness. We need to know that we can cope with schizophrenia and we need to find a way to not isolate ourselves.

[Male, 43 years of age, Cape Town]

